# Efficacy and Safety Analysis of Triple Therapy (Pabolizumab+Cryoablation+Renvastinib) for Patients with Unresectable Hepatocellular Carcinoma (uHCC)

**DOI:** 10.34172/aim.34630

**Published:** 2025-10-01

**Authors:** Juan Lei, Zhonghua Chen

**Affiliations:** ^1^Medical Service Training Center, 900th Hospital of Joint Logistics Support Force, Fuzhou, China; ^2^Department of Radiotherapy Oncology, 900th Hospital of Joint Logistics Support Force, Fuzhou, China

**Keywords:** Cryoablation, Immunotherapy, Renvastinib, Triple therapy, Unresectable hepatocellular carcinoma

## Abstract

**Background:**

To determine whether employing a monoclonal antibody against programmed death receptor-1 (PD-1) improves the safety and effectiveness of cryoablation used with Renvastinib to treat unresectable hepatocellular carcinoma (uHCC).

**Methods:**

Our study retrospectively enrolled 232 uHCC patients who were treated at our medical center between January 2019 and December 2023. Propensity score matching (PSM) was employed in this study for 1:1 matching, and 86 patients were matched in each group. Following matching, the two groups’ negative events, and assessments were made on the objective response rate (ORR), disease control rate (DCR), progression-free survival (PFS), and overall survival (OS). When comparing two groups, a group *t* test was employed to determine whether the quantitative data were normally distributed. The two groups’ survival rates were calculated using the Kaplan-Meier method, survival curves were made, and the log-rank test was performed to find differences between the two groups.

**Results:**

The median follow-up period was 28 months. Forty deaths (46.0%) happened in the double group, whereas 33 deaths (38.0%) occurred in the triple group. The ORR and DCR of the triple treatment group were significantly higher than those of the double therapy group (ORR: 35.6% vs. 14.5%, *P*=0.08; DCR: *P*=0.003; 86.1% vs. 64.1%). Compared to the double group, the OS and PFS rates in the triple group were considerably higher (*P*=0.045 and *P*=0.026, respectively). Analysis of univariate and multivariable Cox risk proportional models showed that AFP level (HR=2.37, *P*=0.001) and treatment regimen (HR=0.60, *P*=0.38) were independent risk factors for OS. Independent risk variables for PFS included diabetes mellitus (HR=1.94, *P*=0.05), prior local treatment (HR=0.63, *P*=0.014), treatment protocol (HR=0.65, *P*=0.025), and distant metastasis (HR=0.58, *P*=0.09). The incidence of negative reactions did not differ significantly between the two groups (*P*>0.05).

**Conclusion:**

Compared with cryoablation combined with renvatinib, cryoablation combined with renvatinib and PD-1 mAb significantly improved the efficacy and survival of patients with uHCC without increasing adverse events, giving unresectable liver cancer a clinical foundation for treatment optimization.

## Introduction

 Hepatocellular carcinoma (HCC) ranks among the top causes of death among cirrhosis patients.^1^ However, only 15% of liver cancer patients receive radical treatment. Therefore, exploring treatment strategies for unresectable hepatocellular carcinoma (uHCC) is key to improving patient prognosis.^2-4^ Common local treatments include radiofrequency ablation, microwave ablation, cryoablation, and interventional therapy.^5-7^ Among these treatments, cryoablation has been increasingly used because of its advantages, such as minimal damage to large blood vessels, reduced risk of vascular complications, low incidence of pain and controllable formation of ice balls.^8^ Cryoablation can also produce “ectopic tumor suppression” so that local treatment and immunotherapy can achieve a more lasting effect, providing a theoretical basis for combining cryoablation with targeting and immunotherapy.^9-11^

 In recent years, systemic therapies for liver cancer, such as sorafenib, Renvastinib and donafenib, have been recommended as first-line therapies for uHCC. The FDA has authorized attilizumab, a PD-L1 monoclonal antibody, for treatment of uHCC. Although targeted therapy and immunotherapy play important roles in controlling liver cancer progression, the objective response rate (ORR) is still less than 30%, and the ORR of the first-line treatment for HCC, bevacizumab combined with PD-L1 (T + A), is only 27%.^12^ Compared with treatment with targeted drugs alone, transhepatic arterial chemoembolization (TACE) combined with targeted drugs extends the median total survival (OS) and median progression-free survival (PFS) of patients (*P* < 0.05). Studies have also shown that immune checkpoint inhibitors combined with ablation therapy increase the median OS of patients with liver cancer (P < 0.05).^13^ It can be concluded that for uHCC patients, local ablation combined with targeted or immunodrug therapy can improve survival. In addition, local cryoablation, as an important local therapy, has the unique advantage of inducing a specific tumor immune response by releasing tumor-specific antigens.^14,15^ Therefore, immunotherapy on the basis of cryoablation therapy creates conditions for achieving a durable and enhanced antitumor immune response. Nevertheless, it is unknown if local cryoablation in conjunction with renvastinib and PD-1 monoclonal antibodies is safe and effective.

## Materials and Methods

###  Research Subjects

 This study included uHCC patients treated at our medical center from January 2019 to December 2023.

 Inclusion criteria: (1) According to the American Academy of Hepatology’s practice guidelines, patients with uHCC who have been diagnosed by imaging (enhanced CT/MRI or pathological biopsy) and are undergoing cryoablation in conjunction with lenvatinib (double combination); (2) Child‒Pugh Grade A or B (score ≤ 7 points); (3) BCLC stage B or C; and (4) a physical condition score (ECOG PS) of 0~1.

**Figure 1:**
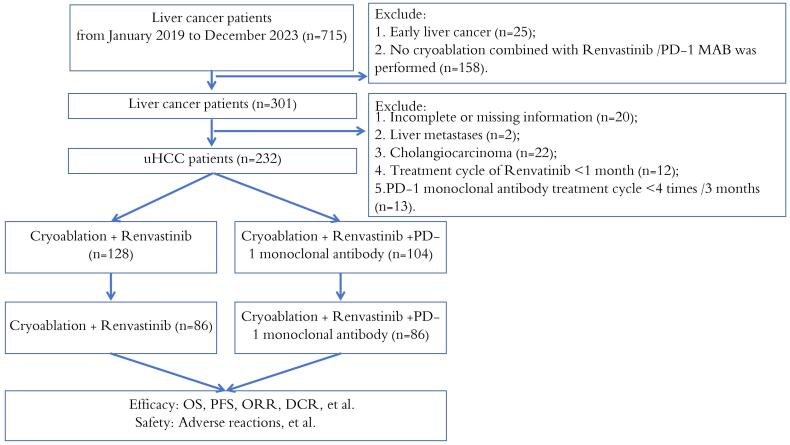
Patient Enrollment Flow Chart

 Exclusion criteria: (1) Having hepatic metastases or cholangiocarcinoma; (2) A treatment cycle of lenvatinib of less than 1 month; (3) Less than 4 PD-1 monoclonal antibodies; (4) Severe comorbidities, including severe heart, lung, kidney or coagulation dysfunction and other uncontrolled chronic diseases and mental disorders; (5) HIV infection; (6) Pregnant women or children; (7) Incomplete clinical data (Figure 1).

###  Research Methods

 Cryoablation procedure: Using a cryo-probe guided by CT and the cryo-care system (Endocare), a twofold freezing-thawing cycle was performed. The double freeze‒thaw cycle consisted of 20 minutes of freezing, followed by 10 minutes of thawing and 15 minutes of freezing, and the freezing probe temperature was reduced to -165 ± 2 °C within 1 minute. To prevent the ablation from spreading to nearby vital structures, real-time ultrasound monitoring of the ablation procedure was undertaken. The hemostatic gelatin sponge was inserted into the sheath, the hemostatic sponge was plugged, and the sheath was removed.

 Depending on body mass. PD-1 monoclonal antibody (200 mg) was intravenously administered once every 3 weeks, and the drug dose was adjusted according to the manufacturer’s instructions. The revised Solid Tumor Response Assessment Criteria (mRECIST) were used every 8-12 weeks to assess patient response to therapy.

###  Curative Effect Evaluation

 OS is defined as the period of time from the beginning of treatment and the last follow-up or death (for any reason). PFS is defined as the interval between the initiation of treatment and the tumor’s progression (in any manner), death (for any cause), or the most recent follow-up, whichever came first.

 Complete response (CR), partial response (PR), stable disease (SD), progressive disease (PD), objective response rate (ORR), and disease control rate (DCR) were among the mRECIST criteria used to evaluate efficacy.

###  Safety Evaluation

 CTCAE version 5.0 was used to analyze adverse events. Level 1: Mild; asymptomatic or mild; clinical or diagnostic findings only; no treatment needed. Level 2: Moderate; requiring minor, local or noninvasive treatment; Age-appropriate restriction of instrumental activities of daily living. Level 3: Severe or medically significant but not immediately life-threatening; leading to hospitalization or extended hospitalization; being incapacitated; and limiting one’s ability to undertake self-rational daily living activities. Level 4: Potentially fatal; immediate medical care is required. Level 5: Death as a result of an adverse incident.

###  Follow-up Visit

 The last follow-up ended on March 31, 2024, and the efficacy follow-up was regularly performed every 3 months after the initial treatment. Thorough medical history and physical examination were part of every follow-up, as well as hematological and biochemical tests, enhanced abdominal CT or MRI, chest CT and other imaging tests, and laboratory tests, including PLT, liver function, AFP, etc. During follow-up, treatment could be discontinued if there was an intolerable toxic reaction, tumor progression, or a change in the treatment plan. On the basis of the results of the multidisciplinary panel discussion and the requirements of the patient, the choice of subsequent treatment, such as second-line targeted agents, PD-1 inhibitors (for patients receiving dual therapy), radiotherapy (including 125I particle brachytherapy), TACE, hepatic arterial infusion chemotherapy (HAIC), or optimal supportive treatment, was determined.

###  Statistical Analysis

 SPSS 26, GraphPad Prism 9 and R 4.0 were used for data analysis. To avoid selection bias in retrospective cohort studies, propensity score matching (PSM) (0.2 of the SD of the logit of the PS)^16^ was used. By using the logit model, factors that may affect treatment efficacy were identified, including ECOG status, age, sex, BMI, disease etiology, baseline liver function, degree of portal vein cancer thrombus, baseline AFP level, previous treatment, BCLC grade, Child grade, portal hypertension, and the presence or absence of distant metastasis.

 The minimum fully confounding variable set was identified and selected through a causal directed acyclic graph (cDAG) to ensure the adjustment of all potential confounding factors. Normally distributed quantitative data are represented as mean ± SD. The balance of confounding factors in the matched population should be evaluated using the standardized mean difference (SMD) (SMD < 0.1).^17^

## Results

###  Analysis of Clinical Medical Records

 In total 232 patients were part of the PSM analysis, and after matching, 172 comparable HCC patients (1:1 matching) were selected for analysis, including 86 patients in either the triple and double groups (Figure 1). After matching, the difference between the two groups was reduced, and the factors included in the analysis were balanced in both groups (*P* value > 0.05). In the overall cohort after PSM, the proportion of patients < 60 years old was greater than that of patients ≥ 60 years old, and the proportion of men was higher. A total of 157 patients (91.2%) were infected with HBV alone, 93.0% and 89.5% were in the triple group and the double group, respectively, and only 2 patients had no history of cirrhosis. Most of the enrolled patients had multiple lesions, including 74 patients (86%) with triple combination and 69 patients (80.2%) with double combination. Most of the patients had vascular invasion, including 55 patients (64.0%) with triple combination and 58 patients (67.4%) with double combination. There were 34 patients (39.5%) and 28 patients (32.6%) with distant metastasis in the two groups (double and triple). There were 124 patients (73%) with stage C BCLC, 60 patients (69.8%) in the triple group and 64 patients (74.4%) in the double group. Most of the patients had Child‒Pugh stage A disease, including 55 patients (64.0%) in the triple group and 60 patients (69.8%) in the double group. Following matching, the majority of patients had already undergone cancer-related treatment (Table 1).

**Table 1:** Clinical Features before and after PSM in the Cryo + Lenvatinib + anti-PD-1 Group and Cryo + Lenvatinib Group

**Project**	**Before PSM**					**After PSM**			
	**Total (n=232)**	**Triple (n=104)**	** Double (n=128)**	**Statistical value**	***P*** ** value**	**Triple (n=86)**	**Double (n=86)**	** Statistical value**	***P*** ** value**
Gender [ (%)]				χ^2^ = 0.000	0.987			χ^2^ = 0.657	0.418
Female	20 (8.6)	9 (8.7)	11 (8.6)			9 (10.5)	6 (7.0)		
Male	212 (91.4)	95 (91.3	117 (91.4			77 (89.5)	80 (93.0		
Age [ (%)]				χ^2^ = 0.100	0.752			χ^2^ = 0.102	0.749
< 60 years old	152 (65.5)	67 (64.4)	85 (66.4)			55 (64.0)	57 (66.3)		
≥ 60 years old	80 (34.5)	37 (35.6	43 (33.6)			31 (36.0)	29 (33.7)		
BMI [ (%)]				χ^2^ = 0.525	0.469			χ^2^ = 0.024	0.878
< 24 kg/m^2^	102 (44.0)	43 (41.3)	59 (46.1)			37 (43.0)	38 (44.2)		
≥ 24 kg/m^2^	130 (56.0)	61 (58.7)	69 (53.9)			49 (57.0)	48 (55.8)		
Portal hypertension [ (%)]				χ^2^ = 0.009	0.926			χ^2^ = 0.000	> 0.05
Yes	188 (81.0)	84 (80.8)	104 (81.2)			70 (81.4)	70 (81.4)		
No	44 (19.0)	20 (19.2)	24 (18.8)			16 (18.6)	16 (18.6)		
Etiology [ (%)]				χ^2^ = 3.561	0.169			χ^2^ = 0.657	0.72
HBV	206 (88.8)	96 (92.3	110 (85.9)			80 (93.0)	77 (89.5)		
HCV	13 (5.6)	2 (1.9)	9 (7.0)			2 (2.3)	3 (3.5)		
Other	19 (8.2)	6 (5.8)	9 (7.0)			4 (4.7)	6 (7.0)		
Antivirus [ (%)]				χ^2^ = 2.740	0.098			χ^2^ = 1.758	0.185
Yes	190 (81.9)	90 (86.5)	100 (78.1)			72 (83.7)	65 (75.6)		
No	42 (18.1)	14 (13.5)	28 (21.9)			14 (16.3)	21 (24.4)		
AFP [ (%)]				χ^2^ = 9.316	0.002			χ^2^ = 0.595	0.440
< 400 ng/mL	126 (54.3)	68 (65.4)	58 (45.3)			52 (60.5)	47 (54.7)		
2400 ng/mL	106 (45.7)	36 (34.6)	70 (54.7)			34 (39.5)	39 (45.3)		
PLT ( × 10^9^/L)	125 (90.0~187.0)	118 (80.0146.0)	142 (96.2~203.8)	U = 5183 0.002	0.002	119.5 (88.8~155.8)	133 (92.8~203.3)	U = 3180	0.115
ALT (U/I)	35 (22.0~57.0)	37 (24.0~74.5)	33 (19.5~53.1)	U = 5826 0.121	0.121	36 (23.5~55.3)	33.5 (21.8~59.0)	U = 3530	0.607
AST (U/L)	41 (28.0~77.9)	38 (27.3~78.8)	44 (28.3~77.5)	U = 6583 0.747	0.747	37 (27.0~61.2)	37 (27.0~76.3)	U = 3655	0.896
TBil (μmal/L)	17.5 (12.5~23.6)	18.1 (11.4~25.6)	17.0 (12.8~22.8)	U = 6284	0.702	18 (11.4~25.6)	16.9 (12.3~22.5)	U = 3424	0.401
Child‒Pugh grading [(%)]				χ^2^ = 0.323	0.57			χ^2^ = 0.656	0.418
A-level	154 (66.4	67 (64.4)	87 (68.0)			55 (64.0)	60 (69.8)		
B-level	78 (33.6)	3 (35.6)	41 (32.0)			31 (36.0)	26 (30.2)		
ALBI grading [(%)]				χ^2^ = 1.039	0.595			χ^2^ = 0.089	0.502
Level 1	49 (21.1)	19 (18.3	30 (23.4)			17 (19.8)	22 (25.6)		
Level 2	171 (73.7)	80 (76.9)	91 (71.1)			65 (75.6)	62 (72.1)		
Level 3	12 (5.2)	5 (4.8)	7 (5.5)			4 (4.7)	2 (2.3)		
Number of tumors [(%)]				χ^2^ = 4.247	0.039			χ^2^ = 1.037	0.309
Single	45 (19.4	14 (13.5)	31 (24.2)			12 (14.0)	17 (19.8)		
Multiple	187 (80.6)	90 (86.5)	97 (75.8)			74 (86.0)	69 (80.2)		
Maximum diameter of tumor [(%)]				χ^2^ = 10.918	0.001			χ^2^ = 0.611	0.434
< 7.07 cm	133 (57.3)	72 (69.2)	61 (47.7			55 (64.0)	50 (58.1)		
≥ 7.07 cm	99 (42.7)	32 (30.8	67 (52.3)			31 (36.0)	36 (41.9)		
Vascular invasion [(%)]				χ^2^ = 0.998	0.318			χ^2^ = 0.232	0.630
Yes	153 (65.9)	65 (62.5)	88 (68.8)			55 (64.0)	58 (67.4)		
No	79 (34.1)	39 (37.5)	40 (31.2)			31 (36.0)	28 (32.6)		
Distant metastasis [(%)]				χ^2^ = 0.353	0.553			χ^2^ = 0.908	0.341
Yes	80 (34.5)	38 (36.5	42 (32.8)			34 (39.5)	28 (32.6)		
No	152 (65.5)	66 (63.5)	85 (66.4)	52 (60.5)	57 (66.3)	52 (60.5)	57 (66.3)		
BCIC grading [(%)]				χ^2^ = 1.041	0.308			χ^2^ = 0.497	0.497
B-level	68 (29.3	34 (32.7)	34 (26.6)			26 (30.2)	22 (25.6)		
C-level	164 (70.7)	70 (67.3)	94 (73.4)			60 (69.8)	64 (74.4)		
Diabetes [(%)]				χ^2^ = 1.252	0.263			χ^2^ = 0.154	0.695
Yes	46 (19.8	24 (23.1)	22 (17.2)			17 (19.8)	15 (17.4)		
No	186 (80.2)	80 (76.9)	106 (82.8)			69 (80.2)	71 (82.6)		
Liver cirrhosis [(%)]				χ^2^ = 0.170	0.009			χ^2^ = 2.024	0.155
Yes	224 (96.6)	104 (100.0)	120 (93.8)			86 (100.0	84 (97.7)		
No	8 (3.4)	0 (0.0)	8 (6.2)			0 (0.0)	2 (2.3)		
ECOGPS score [(%)]				χ^2^ = 0.816	0.366			χ^2^ = 0.821	0.312
0	1 (0.4)	0 (0.0)	1 (0.8)			0 (0.0)	0 (0.0)		
1	231 (99.6)	104 (100.0)	127 (99.2)			86 (100.0)	86 (100.0)		
Previous local treatment [(%)]				χ^2^ = 7.118	0.008			χ^2^ = 0.841	0.359
Yes	118 (50.9)	63 (60.6)	55 (43.0)			49 (57.0)	43 (50.0)		
No	114 (49.1)	41 (39.4)	73 (57.0)			37 (43.0)	43 (50.0)		
Previous systematic treatment [(%)]				χ^2^ = 0.704	0.401			χ^2^ = 0.073	0.787
Yes	22 (9.5)	8 (7.7)	14 (10.9)			7 (8.1)	8 (9.3)		
No	210 (90.5)	96 (92.3)	114 (89.1)			79 (91.9)	78 (90.7)		
Cryoablation [(%)]				χ^2^ = 0.000	0.984			χ^2^ = 0.462	0.497
1	69 (29.7)	31 (29.8)	38 (29.7)			26 (30.2)	22 (25.6)		
> 1	163 (70.3)	73 (70.2)	90 (70.3)			60 (69.8)	64 (74.4)		

###  Comparison of OS and PFS between the Two Groups

**Figure 2:**
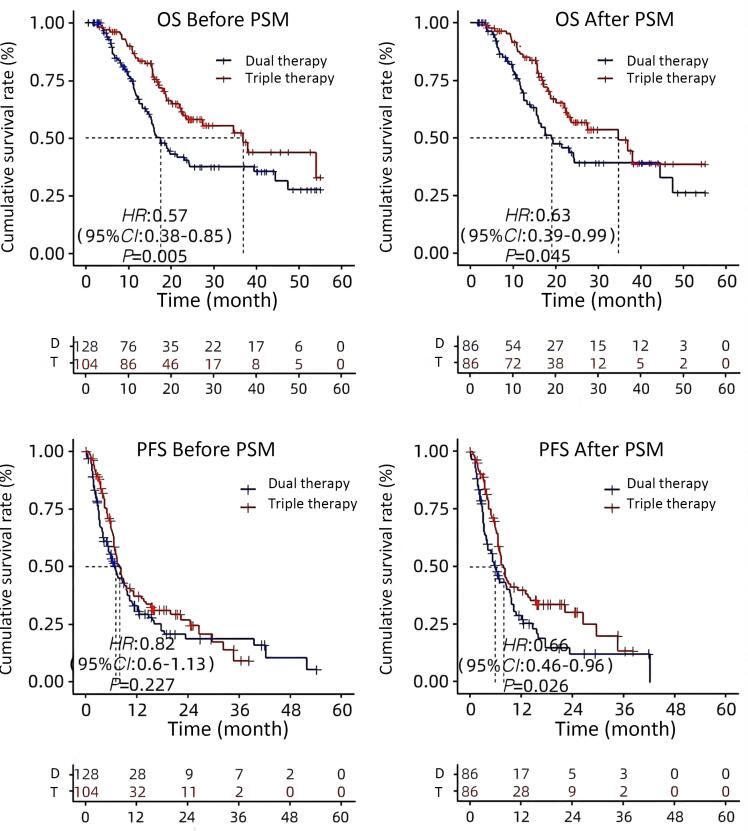
OS and PFS Comparisons before and after PSM

 There were 40 deaths (46.0%) in the double group and 33 deaths (38%) in the triple group with a median follow-up of 28 months. The survival rate was considerably greater for patients in the triple group than for those in the double group. The OS and PFS of patients in the triple group were 34.6 months and 8.0 months. The OS and PFS in the double group were 19.0 months and 6.0 months. The OS and PFS of the two groups differed statistically significantly (*P* values: 0.045 and 0.026, respectively) (Figure 2).

###  Cox Regression Analysis after PSM

 The treatment regimen and AFP level were found to be independent factors affecting OS (*P* < 0.05). PFS was observed to be independently influenced by treatment regimen, diabetes mellitus status, prior local treatment, and distant metastasis (all *P* < 0.05) (Table 2). Cox regression analysis before PSM revealed that treatment regimen, AFP level, previous local treatment, and number of cryoablations were independent factors influencing OS (*P* < 0.05) (Table 3). Cox regression analysis before and after PSM revealed that the treatment regimen and AFP level were independent influencing factors for OS.

**Table 2:** Single Factor Regression Analysis of Factors Influencing OS and PFS after PSM

**Item**	**OS**				**PFS**			
	**Univariate analysis**		**Multivariate analysis**		**Univariate analysis**		**Multivariate analysis**	
	**HR (95% CI)**	***P***	**HR (95% CI)**	***P***	**HR (95% CI)**	***P***	**HR (95% CI)**	***P***
Treatment plan (triple vs double)	0.63(0.39~0.99)	0.047	0.6(0.37~0.97)	0.038	0.66(0.46～0.96)	0.028	0.65 (0.44~0.95)	0.025
Gender (male vs female)	0.73(0.31～1.68)	0.453			1.09(0.60～1.98)	0.782		
Age ( > 60 years vs < 60 years)	0.88(0.54～1.43)	0.615			1.03(0.70～1.51)	0.886		
BMI ( ≥ 24ky/m^2^ vs < 24 kg/m^2^)	1.49(0.93~2.39)	0.100			1.37(0.94~2.00)	0.099		
Child‒Pugh grading (B vs. A)	0.95(0.58～1.57)	0.842			0.96(0.65～1.42)	0.847		
BCLC grading (C and B)	1.82(1.05～3.14)	0.032	0.86(0.33～2.23)	0.758	0.79(0.54～1.17)	0.234		
Diabetes (Yes or No)	0.7(0.37～1.30)	0.256			1.69(1.08～2.64)	0.022	1.94(1.22～3.09)	0.005
Portal hypertension (Yes or No)	2.08(1.03～4.20)	0.042	1.73(0.83～3.58)	0.142	0.89(0.57～1.41)	0.625		
Cirrhosis (Yes or No)	20.72(0.01～5207.00)	0.448			0.81(0.20～3.27)	0.763		
HBV (Yes or No)	0.89(0.38～2.05)	0.776			0.88(0.45~1.75)	0.722		
Antiviral (Yes or No)	0.93(0.52～1.63)	0.789			0.94(0.59~1.48)	0.779		
Number of tumors ( > 1 vs 1)	0.96(0.54～1.73)	0.901			1(0.61～1.62)	0.988		
Tumor size ( ≥ 7.07 cm vs < 7.07 cm)	1.68(1.05~2.68)	0.030	1.18(0.70～1.98)	0.537	0.76(0.52～1.12)	0.16		
AFP ( > 400 ng/mL vs < 400 ng/mL)	2.46(1.55~3.92)	≤ 0.001	2.37(1.44～3.88)	0.001	1.06(0.73～1.53)	0.772		
Previous local treatment (Yes or No)	0.55(0.34～0.87)	0.012	0.67(0.41～1.11)	0.123	0.63(0.44～0.91)	0.013	0.63(0.43～0.89)	0.014
Previous systematic treatment (Yes or No)	0.64(0.26～1.58)	0.331			0.74(0.39～1.42)	0.372		
Follow up local treatment (Yes or No)	0.41(0.10～1.70)	0.220			1.8(0.87～3.72)	0.110		
Subsequent systematic treatment (Yes or No)	0.39(0.16～0.97)	0.042	0.62(0.24～1.61)	0.325	1.17(0.72～1.89)	0.532		
Cryoablation frequency ( > 1 vs 1)	0.79(0.47～1.33)	0.382			1.56(0.99~2.45)	0.054		
Distant metastasis (Yes or No)	1.16(0.72～1.86)	0.539			0.53(0.35～0.80)	0.002	0.58 (0.38~0.87)	0.009
Vascular invasion (Yes or No)	1.86(1.11~3.12)	0.019	1.79(0.73～4.41)	0.204	0.89(0.61～1.30)	0.553		

**Table 3:** Multifactor Regression Analysis of Factors Influencing OS and PFS before PSM

**Project**	**OS**				**PFS**			
	**Univariate analysis**		**Multivariate analysis**		**Univariate analysis**		**Multivariate analysis**	
	**HR (95% CI)**	***P***	**HR (95% CI)**	***P***	**HR (95% CI)**	***P***	**HR (95% CI)**	***P***
Treatment plan (triple vs double)	0.57(0.38~0.85)	0.005	0.63(0.41～0.98)	0.039	0.82(0.60～1.13)	0.228	0.77(0.54～1.10)	0.149
Gender (male vs female)	0.86(0.42～1.78)	0.693			1.19(0.70～2.02)	0.526		
Age (260 years vs. < 60 years)	0.83(0.55~1.26)	0.379			0.9(0.64~1.25)	0.520		
BMI ( ≥ 24 kg/m^2^ vs < 24 kg/m^2^)	1.31(0.88～1.96)	0.179			1.24(0.90~1.70)	0.190		
Child‒Pugh grading (B vs. A)	1.22(0.81～1.84)	0.335			0.99(0.71~1.38)	0.946		
BC LC classification（C vs. B）	1.69(1.07～2.65)	0.024	0.92(0.41~2.07)	0.846	0.79(0.57～1.10)	0.171	0.61(0.32~1.16)	0.132
Diabetes (Yes or No)	0.72(0.43~1.22)	0.227			1.44(0.98～2.12)	0.062		
Portal hypertension (Yes or No)	1.72(0.99~3.00)	0.053			0.89(0.60～1.31)	0.549		
Cirrhosis (Yes or No)	1.12(0.41～3.06)	0.819			1.04(0.46～2.35)	0.929		
HBV (Yes or No)	1.08(0.56～2.07)	0.826			0.97(0.58～1.60)	0.894		
Antiviral (Yes or No)	0.77(0.48～1.24)	0.285			0.86(0.57～1.29)	0.464		
Number of tumors ( > 1 vs 1)	0.85(0.53~1.35)	0.487			1.09(0.73～1.62)	0.682		
Tumor size ( > 7.07 cm vs < 7.07 cm)	1.83(1.24~2.71)	0.002	1.09(0.71~1.68)	0.689	0.76(0.55~1.05)	0.100	0.71(0.49～1.02)	0.064
AFP ( ≥ 400 ng/mL vs < 400 ng/mL)	2.11(1.42~3.12)	< 0.001	1.75(1.16~2.64)	0.008	0.94(0.69~1.29)	0.709	1(0.72～1.40)	0.989
Previous local treatment (Yes or No)	0.45(0.30～0.68)	< 0.001	0.58(0.38~0.90)	0.014	0.84(0.61～1.15)	0.274	0.78(0.56～1.09)	0.152
Previous systematic treatment (Yes or No)	0.67(0.32～1.37)	0.270			0.91(0.55～1.50)	0.701		
Follow up local treatment (Yes or No)	0.26(0.06～1.08)	0.064			1.63(0.88～3.01)	0.120		
Subsequent systematic treatment (Yes or No)	0.41(0.20～0.84)	0.015	0.66(0.30~1.42)	0.284	1.16(0.77～1.74)	0.489	1.18(0.74～1.86)	0.491
Cryoablation frequency ( > 1 vs 1)	0.62(0.41~0.95)	0.028	0.6(0.39~0.93)	0.023	1.43(0.97～2.10)	0.070	1.35(0.91~2.00)	0.135
Distant metastasis (Yes or No)	0.9(0.60～1.38)	0.661			1.12(0.81~1.56)	0.498		
Vascular invasion (Yes or No)	1.79(1.15~2.76)	0.009	1.73(0.78～3.82)	0.178	0.9(0.65～1.25)	0.523	1.42(0.74～2.71)	0.292

###  Comparison of Tumor Response between the Two Groups

 Compared to the double group, the triple group’s ORR (35.6% vs 14.5%) and DCR (86.1% vs 64.1%) were considerably higher (*P* = 0.08 and 0.0003, respectively). The triple group had lower PD (18.3% vs. 36.0%) and higher PR (25.6% vs. 11.6%) than the double group; these differences were statistically significant (*P* values were 0.0003 and 0.019, respectively) (Table 4).

**Table 4:** Tumor Response

**Evaluation of tumor efficacy**	** Before PSM**			**After PSM**		
	**Triple **	**Double **	***P***	**Triple **	**Double **	***P***
CR [(%)]	9(8.7)	5(3.9)	0.131	7(8.1)	4(4.7)	0.350
PR [(%)]	27(26.0)	13(10.2)	0.002	22(25.6)	10(11.6)	0.019
SD [(%)]	50(48.1)	66(51.6)	0.597	43(50.0)	41(47.7)	0.760
PD [(%)]	18(17.3)	44(34.4)	0.003	14(18.3)	31(36.0)	0.003
ORR [(%)]	36(34.6)	14(14.1)	0.000	29(35.6)	14(14.5)	0.008
DCR [(%)]	86(82.7)	84(65.6)	0.003	72(86.1)	55(64.1)	0.003

###  Subgroup Analysis

**Figure 3:**
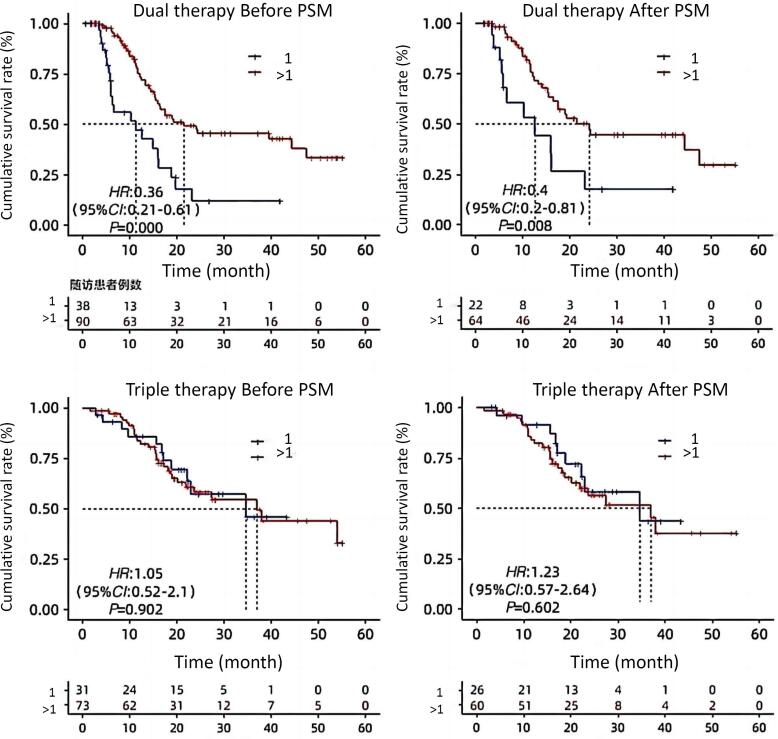
OS Comparison of the Number of Patients with 1 or More Cases of Cryoablation before and after PSM

**Figure 4:**
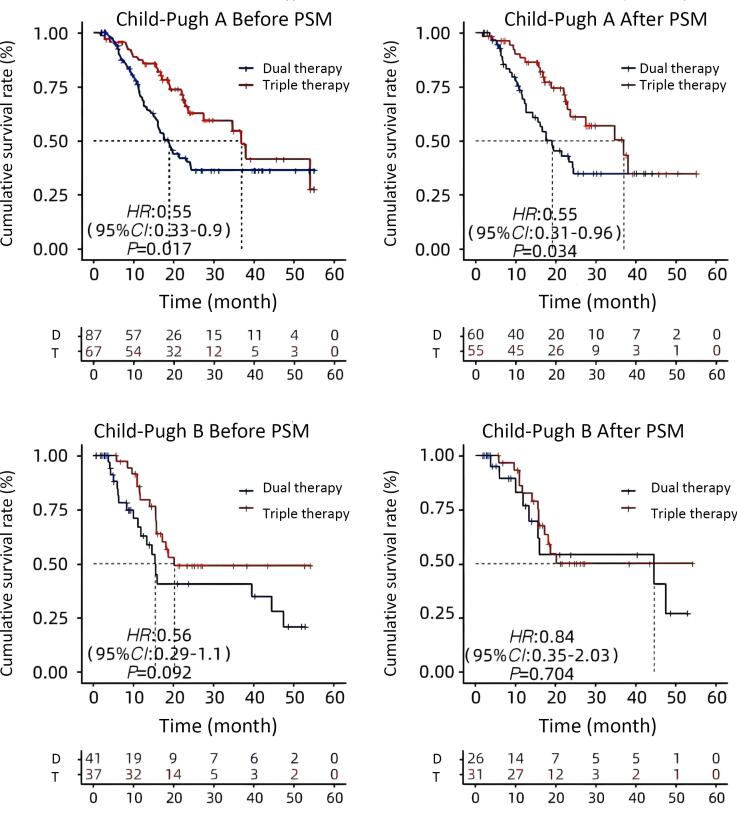
OS Comparison in Individuals with Child-Pugh Class A or B Disease before and after PSM

**Figure 5:**
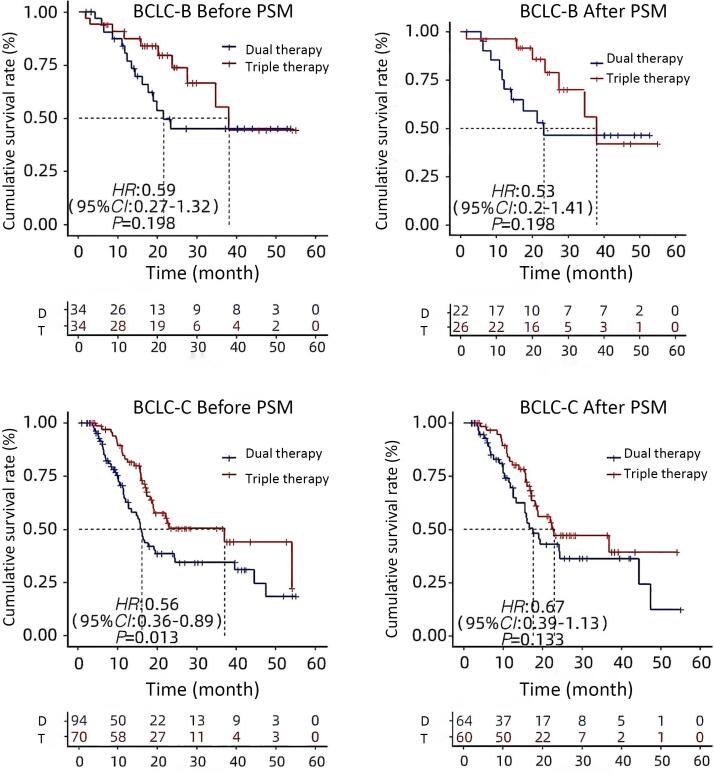
OS Comparison in BCLC-B or BCLC-C Patients before and after PSM

**Figure 6:**
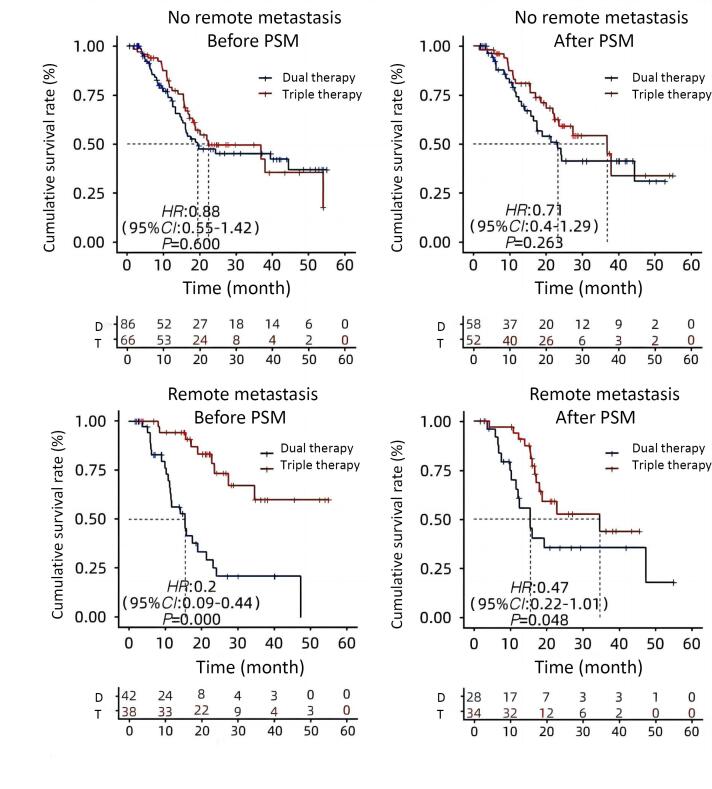
OS Comparison with or without Distant Metastases Prior to and Following PSM

 The double group’s patient OS was extended by receiving more than one cryoablation (24.2 months vs. 12.6 months, *P* = 0.08) (Figure 3). The median overall survival (OS) for Child-Pugh grade A patients were considerably longer in the triple group than in the double group (36.97 months vs. 17.55 months, *P* = 0.034) (Figure 4). The median OS for patients with BCLC stages B and C did not change significantly between triple therapy and double therapy (P values were 0.198 and 0.133, respectively) (Figure 5). The triple group had a considerably longer median OS (34.6 months vs. 15.5 months, *P* = 0.048) than the double group for patients with distant metastases (Figure 6).

## Discussion

 Triple therapy consisting of cryoablation plus lenvatinib plus PD-1 mAb may be a better treatment option for patients with advanced HCC. It can also inhibit hypoxia-induced angiogenesis, regulate the tumor immune microenvironment after cryoablation, and enhance the immune response to PD-1 monoclonal antibodies in HCC.^18-20^ Thus, lenvatinib, PD-1 mAb, and cryoablation together may have a synergistic anticancer effect and improve the clinical prognosis of patients.^21^

 In this study, PD-1 monoclonal antibodies in uHCC patients improved the efficacy of cryoablation combined with renvastinib.^22-24^ Compared with those in the double group, the median PFS was improved, and the ORR and DCR were also significantly increased.^25^ Several other studies of local combined system therapy have also shown promising results, and previous studies have evaluated the safety and efficacy of sorafenib combined with cryoablation for advanced HCC.^26-28^ The results revealed that the median time to disease progression (TTP) was 9.5 months (95% CI: 8.4 ~ 13.5) in the combined treatment group and 5.3 months (95% CI: 3.8 ~ 6.9) in the sorafenib group (*P* = 0.02). The median OS was 12.5 months (95% CI: 10.6–16.4) in the combined treatment group and 8.6 months (95% CI: 7.3–10.4) in the sorafenib group (*P* = 0.01). Compared to the control group, patients with high microvascular density (MVD) in the combination therapy group had much longer median TTPs and OS. Other studies evaluated the combined treatment of TACE, renvastinib, and PD-1 inhibitors in uHCC patients, with PFS ranging from 5.2 to 25.2 months and OS ranging from 12.8 to 35.1 months, which was roughly similar to the efficacy of the cryoablation + renvastinib + PD-1 monoclonal antibody protocol in this study. In a study of patients with portal vein cancer thrombus combined with HCC, the ORR in the TACE-HAIC combined with immunotherapy group was significantly greater than that in the TACE group (53.7% vs 7.8%, *P* < 0.001), and the OS in the combination group was significantly greater than that in the TACE group (*P* < 0.001). Compared with TACE alone, TACE + cryoablation significantly improved survival in group B (11.0 months vs. 6.0 months, *P* = 0.08), as did TACE + cryoablation in group C (8.0 months vs. 5.0 months, *P* = 0.0001). Most of these studies excluded patients with portal vein invasion, distant metastasis or hepatic decompensation, and some included only patients with stage A BCLC. However, the large tumor load in this study (the maximum tumor diameter was 25.3 cm, and most of the tumors had portal vein infiltration or extrahepatic metastasis) may have led to limited survival benefits for these uHCC patients after treatment, and the improvement in PFS was not obvious.^29-31^ However, in this study, some patients had previously received interventional therapy, local therapy, surgery, other systemic therapy, chemoradiotherapy, or sequential therapy, so the median OS was significantly improved.^32-34^ Compared to patients receiving double therapy, those with advanced HCC who received triple therapy had a considerably higher survival rate.

 According to further subgroup analysis, the median OS of patients with Child‒Pugh grade A HCC with distant metastasis was significantly longer in the triple group than in the double group. OS was prolonged by > 1 case of cryoablation in the double group.^35^ Therefore, for patients with Child‒Pugh grade A disease and distant metastasis, combining PD-1 monoantibodies with cryoablation and renvastinib is necessary. Adverse events in all triads were manageable and were approximately the same as those reported in previous studies of HCC, with no new or unexpected adverse events observed. In addition, the incidence and severity of adverse events in the triple group were similar to those in the double group without significant differences, suggesting that cryoablation plus renvastinib plus PD-1 mAb did not significantly increase the risk of adverse events, suggesting that the triple regimen had an acceptable safety profile.^36^

 This was a retrospective study conducted in a single medical center and limited in sample size, which may have led to some inherent selection bias, all of which cannot be completely eliminated by PSM analysis.^37^ Other confounding prognostic factors (such as comorbidities and socioeconomic status) may be present, as well as imperfect matches. Therefore, it is necessary to validate the conclusions of this study through further randomized trials.^38-40^

 In conclusion, this study found that advanced HCC could be safely and effectively treated using cryoablation plus renvastinib plus a PD-1 monoclonal antibody. Patients treated with cryoablation plus renvastinib plus an anti-PD-1 mAb showed a better treatment response and improved survival than patients treated with cryoablation plus renvastinib.

## Conclusion

 Unresectable liver cancer now has a clinical basis for therapy optimization thanks to cryoablation combination with Renvastinib and PD-1 mAb, which dramatically increased the efficacy and survival of patients with uHCC without increasing adverse effects.
